# Four common polymorphisms of BRIP1 (rs2048718, rs4988344, rs4986764, and rs6504074) and cancer risk: evidence from 13,716 cancer patients and 15,590 cancer-free controls

**DOI:** 10.18632/aging.101388

**Published:** 2018-02-16

**Authors:** Di Liu, Yi Zheng, Meng Wang, Yujiao Deng, Shuai Lin, Linghui Zhou, Pengtao Yang, Cong Dai, Peng Xu, Qian Hao, Dingli Song, Huafeng Kang, Zhijun Dai

**Affiliations:** 1 Department of Oncology, the Second Affiliated Hospital of Xi’an Jiaotong University, Xi’an710004, China; * Equal contribution

**Keywords:** BRIP1, polymorphism, cancer risk, meta-analysis

## Abstract

Previous studies have showed the associations between various BRCA1-interacting protein 1 (*BRIP1*) polymorphisms and cancer risk. But, these results were inconsistent. This meta-analysis based on 18 studies involving 13,716 cancer patients and 15,590 cancer-free controls is aimed at to evaluate the relationship between the four common SNPs of *BRIP1* (rs2048718, rs4988344, rs4986764, and rs6504074) and cancer risk. The results showed a decreased risk of rs2048718 or rs4986764 for cervical cancer rather than breast cancer in the overall population (*P* < 0.05). However, rs6504074 was associated with gynecologic cancer risk among overall population (*P* < 0.05). Further stratification analyses by ethnicity indicated that all 4 polymorphisms (rs2048718, rs4988344, rs4986764, and rs6504074) were strongly related to cancer susceptibility in Chinese people (*P* < 0.05). This meta-analysis showed that rs6504074 may play a decreased risk of gynecologic cancer in the overall population. Rs4988344, rs4986764, and rs6504074 were significantly related to decreasing cancer risk in Chinese population.

## Introduction

The germ-line breast cancer 1 interacting protein 1 (*BRIP1*) comes to light as a crucial protein for BRCA1-dependent DNA damage repair functions [1-3]. The human *BRIP1* gene (also named *FANCJ* or *BACH1*) is located on chromosome 17q22, comprising of 19 introns and 20 exons, and encodes BRCA1-associated C-terminal helicase 1 [1,4] And its mutations that affect helicase activity have been identified in patients suffering early-stage breast cancer. Missense mutations in *BRIP1* may increase breast cancer risk [5]. Therefore, it is considered as a moderate-penetrance susceptibility gene for breast cancer. However, previous studies declared that *BRIP1* mutation not only has effect on breast cancer, but also in other various cancers including cervical cancer [5-7], ovarian cancer [4,8] and prostate cancer [9].

It is observed that the genetic polymorphisms in BRIP1 influence the cancer susceptibility by altering their natural function. And many single-nucleotide polymorphisms (SNPs) in *BRIP1* have been recognized. SNPs may alter the expression, processing, and transcription of genes, and thus contribute to cancer development. Numerous epidemiological studies have demonstrated that some SNPs located within genes can alter their expression and/or maturation and are associated with cancer susceptibility and progression.

Recently, numerous molecular epidemiology studies explored the relationship between *BRIP1* polymorphisms and cancer susceptibility. Nevertheless, their results were inconclusive. Polymorphisms of *BRIP1* is regarded as an important susceptibility factor in cervical cancer, but not in breast cancer [5-7]. For example, Due to the inconsistencies among these previous studies, we conducted this meta-analysis covering all eligible molecular epidemiology studies to validate the correlation of four most common *BRIP1* polymorphisms (rs2048718, rs4988344, rs4986764, and rs6504074) and cancer risk.

## RESULTS

### Study characteristics

**Figure 1:**
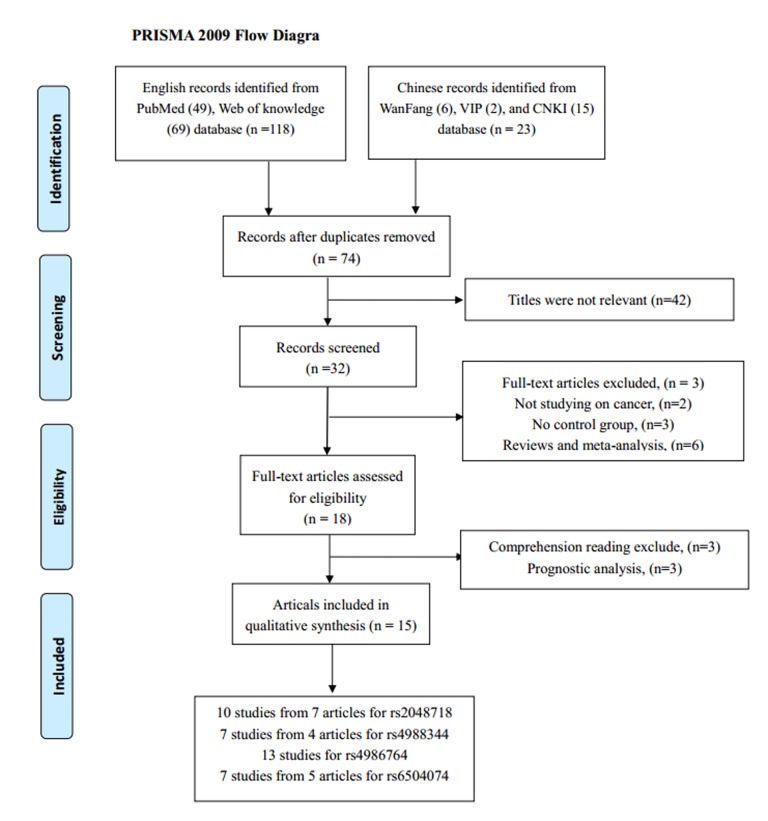
**The flow diagram of the meta-analysis.** CNKI: China National Knowledge Infrastructure.

According to our inclusion criteria, 18 studies from 15 articles containing 13,716 cancer patients and 15,590 cancer-free controls were finally included. The detail screening process was exhibited in Figure 1. It contained four separate studies in Song’s articles focusing on breast cancer and ovarian cancer.

Among the 18 eligible studies, 11 were conducted in Caucasian populations from the UK, Italy, Germany, USA, Canada, Denmark, Cyprus, and Finland. Five studies were on Asian background and all samples were Chinese. And one study was performed on mixed population. All the studies were case-control design, studying on various cancer types including breast, ovarian, cervical, gastric and prostate cancer. Table 1 listed the main characteristics of all included studies.

**Table 1:** Characteristics of the studies included in the meta-analysis.

Study	Year	Country	Ethnicity	Cancer type	Genotyping medthod	Source of control	Case/Control	SNP No.
Carrera-Lasfuentes [20]	2017	Spain	Mixed	GC	QIAamp	Population	598/601	3
Zhou [21]	2014	China	Asian	CC	MassARRAY	Population	309/315	1, 3
Ren [14]	2013	China	Asian	BC	Mass ARRAY	Hospital	319/306	1,2,3,4
Ma 1 [6]	2013	China	Asian	CC	Mass ARRAY	Population	454/562	1,2,3,4
Ma 2 [5]	2013	China	Asian	CC	Mass ARRAY	Hospital	298/286	1,2,3,4
Silvestri [22]	2011	Italy	Caucasian	BC	PCR-SSCP	Hospital	97/203	3
Loizidou [23]	2010	Cyprus	Caucasian	BC	MassArray	Population	1108/1170	3
Huo [24]	2009	China	Asian	BC	PCR-PIRA	Population	568/624	3
Kote-Jarai [9]	2009	UK	Caucasian	PC	TaqMan	Population	1841/1880	4
Guénard [25]	2008	Canada	Caucasian	BC	PCR-RFLP	Hospital	96/70	1,3
Song 1 [4]	2007	UK	Caucasian	BC	TaqMan	Population	2270/2280	1,2
Song 2 [4]	2007	UK	Caucasian	OC	TaqMan	Population	730/855	1,2,4
Song 3 [4]	2007	USA	Caucasian	OC	TaqMan	Population	327/429	1,2,4
Song 4 [4]	2007	Denmark	Caucasian	OC	TaqMan	Population	456/1231	1,2,4
Frank [26]	2007	Germany	Caucasian	BC	TaqMan	Population	571/712	1,3
Garcia-Closas [20]	2006	Mixed	Caucasian	BC	qPCR	Population	1596/1254	3
Vahteristo [27]	2006	Finland	Caucasian	BC	TaqMan	Population	866/731	3
Seal [28]	2006	UK	Caucasian	BC	Pyrosequencing	Population	1212/2081	3

**Abbreviations** BC: breast cancer; CC: cervical cancer; PC: Prostate cancer; OC: ovarian cancer; PCR: polymerase chain reaction; RFLP: restriction fragment length polymorphism. PIRA: PCR-primer introduced restriction analysis; DHPLC, denaturing high -performance liquid chromatography; SSCP: single strand conformation polymorphism; SNP: single-nucleotide polymorphisms; SNP No.1: rs2048718; 2: rs4988344; 3: rs4986764; 4.rs6504074

### Quantitative synthesis of the four polymorphisms

As Table 2 showed, the frequencies of the minor allele (MAF) in the cancer-free controls varied widely across included studies, ranging from 0.23 to 0.62 for rs2048718, 0.14 to 0.62 for rs4988344, 0.26 to 0.60 for rs4986764, and 0.24 to 0.28 for rs6504074. The average frequencies of the minor allele for the four abovementioned polymorphisms were 0.39, 0.35, 0.40, and 0.26, respectively.

**Table 2:** BRIP1 polymorphisms Genotype Distribution and Allele Frequency in this meta-analysis.

Study	Genotype (N)								Allele frequency (N)				MAF	
	Case				Control				Case		Control			HWE
	total	AA	AB	BB	total	AA	AB	BB	A	B	A	B		
**rs2048718**														
Zhou 2014	309	196	94	19	314	183	118	13	486	132	484	144	0.23	0.26
Ren 2013	319	201	98	20	305	177	115	13	500	138	469	141	0.23	0.29
Ma 1 2013	454	285	141	28	560	326	208	26	711	197	860	260	0.25	0.32
Ma 2 2013	298	188	92	18	285	166	106	13	468	128	438	132	0.23	0.45
Guénard 2008	96	38	41	17	70	23	32	15	117	75	78	62	0.41	0.54
Song 1 2007	2170	655	1063	452	2264	655	1151	458	2373	1967	2461	2067	0.47	0.24
Song 2 2007	722	236	341	145	847	246	425	176	813	631	917	777	0.50	0.76
Song 3 2007	322	91	157	74	421	131	195	95	339	305	457	385	0.51	0.17
Song 4 2007	429	118	215	96	1209	352	632	225	451	407	1336	1082	0.62	0.05
Frank 2007	571	181	283	107	712	228	340	144	645	497	796	628	0.49	0.40
**rs4988344**														
Ren 2013	319	65	145	109	306	43	148	115	275	363	234	378	0.62	0.67
Ma 1 2013	454	92	207	155	562	84	270	208	391	517	438	686	0.61	0.81
Ma 2 2013	297	60	136	101	286	42	138	106	256	338	222	350	0.61	0.79
Song 1 2007	2189	1552	585	52	2278	1609	616	53	3689	689	3834	722	0.16	0.51
Song 2 2007	729	498	203	28	848	589	239	20	1199	259	1417	279	0.16	0.46
Song 3 2007	323	228	86	9	427	309	103	15	542	104	721	133	0.16	0.09
Song 4 2007	278	188	82	8	712	526	174	12	458	98	1226	198	0.14	0.58
**rs4986764**														
Carrera-Lasfuentes 2017	598	232	297	69	601	224	270	107	761	435	718	484	0.40	0.11
Zhou 2014	309	164	116	29	315	136	146	33	444	174	418	212	0.34	0.50
Ren 2013	319	168	120	31	306	132	141	33	456	182	405	207	0.34	0.61
Ma 1 2013	454	247	165	42	562	240	258	64	659	249	738	386	0.34	0.67
Ma 2 2013	298	160	110	28	286	122	132	32	430	166	376	196	0.34	0.68
Silvestri 2011	97	37	49	11	203	82	95	26	123	71	259	147	0.36	0.85
Loizidou 2010	1108	465	502	141	1170	475	534	161	1432	784	1484	856	0.37	0.58
Huo 2009	568	308	227	33	624	345	232	47	843	293	922	326	0.26	0.36
Guénard 2008	96	18	48	30	70	12	32	26	84	108	56	84	0.60	0.69
Frank 2007	571	181	295	95	712	226	365	121	657	485	817	607	0.43	0.20
Garcia-Closas 2006	1596	529	761	306	1254	406	612	236	1819	1373	1424	1084	0.43	0.84
Vahteristo 2006	866	184	428	254	731	148	382	201	796	936	678	784	0.54	0.17
Seal 2006	1212	462	549	201	2081	783	970	328	1473	951	2536	1626	0.39	0.34
**rs6504074**														
Ren 2013	319	203	95	21	304	176	110	18	501	137	462	146	0.24	0.88
Ma 1 2013	454	288	137	29	558	317	205	36	713	195	839	277	0.25	0.71
Ma 2 2013	298	188	92	18	284	162	104	18	468	128	428	140	0.25	0.81
Kote-Jarai 2009	1841	963	727	151	1880	1031	727	122	2653	1029	2789	971	0.26	0.68
Song 2 2007	725	417	270	38	847	457	325	65	1104	346	1239	455	0.27	0.50
Song 3 2007	324	170	117	37	421	225	156	40	457	191	606	236	0.28	0.09
Song 4 2007	260	137	104	19	650	340	258	52	378	142	938	362	0.28	0.76

**Abbreviations** A: the major allele, B: the minor allele. MAF: minor allele frequencies.

**Figure 2:**
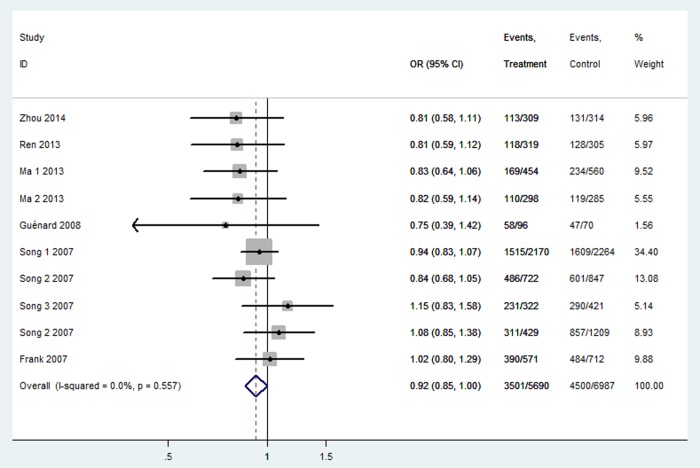
**Forest plot of OR with 95%CI for the BRIP1 polymorphisms with cancer risk under dominant model rs2048718.** CI: confidence interval, OR: odds ratio.

Table 3 listed the results of this meta-analysis. There were 10 studies with 5,690 cancer patients and 6,087 healthy individuals for rs2048718. As displayed in Table 3 and Figure 2, rs2048718 polymorphism had a decrease risk of overall cancer based on the heterozygous and dominant models (AB vs. AA: OR = 0.90, 95% CI = 0.83–0.97, *P* = 0.008; AB+BB vs. AA: OR = 0.92, 95%CI = 0.86–0.99, *P* = 0.037). In the stratified analysis by ethnicity, the results showed significant associations between rs2048718 and cancer risk in the Asian population by heterozygous and dominant comparison (AB vs. AA: OR = 0.76, 95% CI = 0.65–0.89, *P* = 0.001; AB+BB vs. AA: OR = 0.82, 95%CI = 0.70–0.95, *P* = 0.008), while no association was found among Caucasians. However, in the stratified analysis by cancer types, we found a decrease risk between rs2048718 and cervical cancer under heterozygous and dominant model (AB vs. AA: OR = 0.76, 95% CI = 0.64–0.91, *P* = 0.003; AB+BB vs. AA: OR = 0.82, 95%CI = 0.69–0.97, *P* = 0.021), and the results showed no significant difference between rs2048718 polymorphism and gynecologic (breast and ovarian) cancer susceptibility.

**Table 3:** Meta-analysis results.

Comparisons	B vs A		BB vs AA		AB vs AA		BB vs AA+AB		AB+BB vs AA	
	OR (95%CI)	*P*	OR (95%CI)	*P*	OR (95%CI)	*P*	OR (95%CI)	*P*	OR (95%CI)	*P*
rs2048718	0.98(0.93–1.03)	0.408	1.02(0.92–1.14)	0.704	0.90(0.83–0.97)	**0.008**	1.05 (0.96–1.16)	0.269	0.92(0.86–0.99)	**0.037**
Caucasian	0.99(0.89–1.12)	0.921	0.99(0.89–1.12)	0.921	0.95(0.86–1.04)	0.274	1.03 (0.93–1.14)	0.595	0.96(0.88–1.05)	0.381
Asian	0.91(0.81–1.04)	0.160	1.28(0.92–1.80)	0.147	0.76(0.65–0.89)	**0.001**	1.42 (1.02–1.97)	0.040	0.82(0.70–0.95)	**0.008**
CC	0.91(0.79–1.05)	0.209	1.26(0.86-1.85)	0.228	0.76(0.64-0.91)	**0.003**	1.39(0.96–2.03)	0.084	0.82(0.69-0.97)	**0.021**
BC	0.98(0.91–1.05)	0.479	0.98(0.85–1.13)	0.771	0.92(0.82-1.03)	0.151	1.02 (0.90–1.15)	0.820	0.94(0.84–1.04)	0.21
OC	1.01(0.92-1.11)	0.772	1.04(0.86–1.26)	0.665	0.96(0.82-1.11)	0.563	1.07(0.91-1.26)	0.410	0.98(0.85-1.13)	0.775
rs4988344	0.97(0.87–1.09)	0.620	0.89(0.67–1.18)	0.418	0.94(0.80–1.10)	0.441	0.95(0.82-1.10)	0.468	0.93(0.79–1.11)	0.430
Caucasian	1.03(0.93-1.14)	0.533	1.18(0.89–1.57)	0.260	1.03(0.93–1.14)	0.533	1.17(0.88–1.55)	0.282	1.44 (0.99–2.08)	0.38
Asian	0.68(0.54-0.86)	**0.001**	0.66(0.52–0.85)	**0.001**	0.68 (0.54–0.86)	**0.001**	0.88 (0.74–1.04)	0.132	0.78 (0.62–0.97)	**<0.001**
BC	0.92(0.77-1.11)	0.395	0.81(0.51-1.30)	0.391	0.85(0.57-1.25)	0.405	0.93(0.72-1.19)	0.542	0.87(0.56-1.34)	0.519
OC	1.14(0.99-1.30)	0.063	1.41(0.92-2.14)	0.114	1.11(0.94-1.30)	0.211	1.37(0.90-2.09)	0.139	1.63(0.98-2.72)	0.059
**rs4986764**	0.94(0.90-0.98)	**0.001**	0.90(0.82–0.99)	0.024	0.89 (0.80–0.99)	**0.025**	0.95 (0.87–1.03	0.203	0.88(0.80–0.97)	**0.009**
Caucasian	0.99(0.94–1.04)	0.700	0.98(0.88–1.10)	0.779	0.96 (0.89–1.05)	0.361	1.01 (0.92–1.12)	0.781	0.97 (0.90–1.05)	0.426
Chinese	0.81(0.73–0.89)	**0.004**	0.71 (0.56-0.88)	**0.002**	0.73 (0.57–0.93)	**0.011**	0.82(0.66–1.02)	0.072	0.72(0,59-0.90)	**0.004**
CC	0.74(0.65-0.84)	**<0.001**	0.67(0.50-0.89)	**0.006**	0.64 (0.53-076)	**<0.001**	0.83(0.63-1.09)	0.175	0.64 (0.54–0.77)	**<0.001**
BC	0.98(0.93–1.03)	0.406	0.96(0.87–1.07)	0.481	0.96 (0.89–1.03)	0.253	1.00 (0.91–1.09)	0.958	0.96(0.89–1.03)	0.244
**rs6504074**	0.96(0.85–1.09)	0.556	1.04(0.89–1.23)	0.614	0.94 (0.87–1.03)	0.197	1.06 (0.91–1.25)	0.457	0.92(0.79–1.08)	0.298
Caucasian	1.09 (1.00-1.21)	0.059	1.01(0.70–1.44)	0.978	1.02 (0.92–1.12)	0.766	1.01 (0.73–1.40)	0.953	1.08(0.97–1.20)	0.171
Chinese	0.84(0.73–0.97)	**0.016**	0.91(0.64–1.30)	0.610	0.75(0.62–0.89)	**0.002**	1.01(0.71-1.43)	0.939	0.77(0.65–0.91)	**0.003**
OC	1.02(0.87–1.20)	0.787	0.86(0.65–1.13)	0.269	0.95 (0.82–1.11)	0.513	0.87(0.67–1.14)	0.325	1.01 (0.83–1.24)	0.911
GC	0.90(0.82-0.98)	0.015	0.88(0.71-1.09)	0.236	0.86(0.77-0.97)	0.011	0.92(0.75-1.14)	0.463	0.86(0.77-0.96)	0.008

**Abbreviations** A: the major allele; B: the minor allele; CI: confidence interval; OR: odds ratio; GC: gynecologic cancer; BC: breast cancer;OC: ovarian cancer.

There were 7 studies containing 4,589 cancer cases and 5,419 cancer-free controls for rs4988344. As shown in Table 3, The pooled analysis displayed no association of any genetic models and overall cancer risk (All *P* > 0.05). In subgroup analysis by ethnicity, we detected a significant association among Chinese people in four genetic models (B vs. A: OR = 0.68, 95% CI = 0.54–0.86, *P* = 0.001; BB vs. AA: OR = 0.66, 95% CI = 0.54–0.86, *P* = 0.001; AB vs. AA: OR = 0.88, 95% CI = 0.54–0.86, *P* = 0.001; AB+BB vs. AA: OR = 0.78, 95% CI = 0.63–0.97, *P* < 0.001).

**Figure 3:**
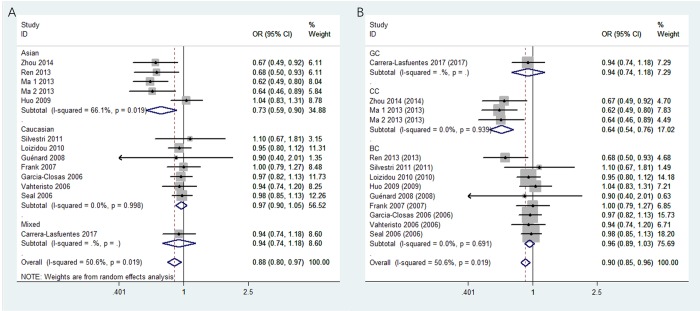
**Stratified analysis based on ethnicity for the association between BRIP1 rs4986764 polymorphism and cancer risk using dominant model.** (**A**) based on ethnicity; (**B**) based on cancer type. CI: confidence interval, OR: odds ratio.

Thirteen studies covering 8,092 cases and 8,915 controls were pooled to evaluate the correlation of rs4986764 and cancer risk. Showing in Table 3 and Figure 3, rs4986764 was associated to reduce cancer risk among the overall population by allele comparison (OR = 0.94, 95% CI = 0.90–0.98, *P* = 0.001), heterozygous comparison (OR = 0.89, 95% CI = 0.80–0.99, *P* = 0.025), and dominant comparison (OR = 0.88, 95% CI = 0.80–0.97, *P* = 0.009). Stratified analysis by ethnicity also displayed significant differences in Chinese population (B vs. A: OR = 0.81, 95% CI = 0.73–0.89, *P* = 0.004; BB vs. AA: OR = 0.71, 95% CI = 0.56–0.88, *P* = 0.005; AB vs. AA: OR = 0.77, 95% CI = 0.57–0.93, *P* = 0.011; AB+BB vs. AA: OR = 0.72, 95% CI = 0.59–0.90, *P* = 0.004). However, there was no significant correlation found in Caucasians for all genetic models (all *P* > 0.05). In the stratified analysis by cancer types, all genetic models failed to detect significant correlations in breast cancer. However, a statistical significance suggested that rs4986764 polymorphism may decrease cervical cancer risk (B vs. A: OR = 0.74, 95% CI = 0.65–0.84, *P* < 0.001; BB vs. AA: OR = 0.67, 95% CI = 0.50–0.89, *P* = 0.006; AB vs. AA: OR = 0.64, 95% CI = 0.53–0.76, *P* < 0.001; AB+BB vs. AA: OR = 0.64, 95% CI = 0.54–0.77, *P* = 0.0001).

A total of 7 studies for rs6504074 included 4,221 cases and 4,944 controls. As shown in Table 3, the overall analysis showed no association between rs6504074 between cancer risk in any genetic models (All *P*﹥0.05). But, further stratification analysis by ethnicity indicated that rs6504074 was significantly associated with a lower cancer risk among Chinese population by three models (B vs. A: OR = 0.84, 95% CI = 0.73–0.97, *P* = 0.016; AB vs. AA: OR = 0.75, 95% CI = 0.62–0.89, *P* = 0.002; BB + AB vs. AA: OR = 0.77, 95% CI = 0.65–0.91, *P* = 0.003). The subgroup analysis by cancer type of rs6504074 also showed a reduced risk for gynecologic cancers in allele model (OR = 0.90, 95% CI = 0.82–0.98, *P* = 0.015), heterozygous model (OR = 0.88, 95% CI = 0.77–0.97, *P* = 0.011), and dominant model (OR = 0.86, 95% CI = 0.77–0.96, *P* = 0.008).

### Heterogeneity analysis and publication bias

The results of the heterogeneity test are displayed in Table 4. When the *P* value of the heterogeneity tests was less than 0.1 (*P* < 0.1), a random effects model was selected. Otherwise a fixed-effect model was applied.

**Table 4:** Heterogeneity-analysis results.

Comparisons	B vs A			BB vs AA			AB vs AA			BB vs AA+AB			AB+BB vs AA		
	I^2^	*P*	EM	I^2^	*P*	EM	I^2^	*P*	EM	I^2^	*P*	EM	I^2^	*P*	EM
rs2048718	0.0%	0.729	F	0.0%	0.679	F	4.6%	0.399	F	0.0%	0.629	F	0.0%	0.557	F
Caucasian	0.0%	0.448	F	0.0%	0.470	F	0.0%	0.998	F	0.0%	0.59	F	0.0%	0.493	F
Chinese	0.0%	1.000	F	0.0%	0.990	F	0.0%	0.572	F	0.0%	0.991	F	0.0%	0.999	F
CC	0.0%	0.998	F	0.0%	0.972	F	0%	0.982	F	0.0%	0.963	F	0.0%	0.993	F
BC	0.0%	0.805	F	0.0%	0.682	F	0%	0.443	F	0.0%	0.525	F	0.0%	0.641	F
OC	45.0%	0.162	F	42.0%	0.179	F	27%	0.253	F	11.5%	0.323	F	42.1%	0.178	F
rs4988344	55.5%	0.036	R	52.9%	0.047	R	55.0%	0.038	R	5.6%	0.384	F	63.3%	0.430	R
Caucasian	27.7%	0.246	F	15.8%	0.312	F	6.7%	0.359	F	11.7%	0.334	F	89.9%	0.000	R
Chinese	0.0%	0.975	F	0.0%	0.963	F	0.0%	0.964	F	0.0%	0.994	F	0.0%	0.428	F
BC	55.6%	0.134	R	59.0%	0.118	R	67.5%	0.080	R	0.0%	0.512	F	74.8%	0.046	R
OC	0.0%	0.404	F	12.6%	0.319	F	0.0%	0.374	F	14.0%	0.312	F	89.0%	0.000	R
rs4986764	45.9%	0.036	F	6.8%	0.378	F	52.8%	0.013	R	12.2%	0.323	F	50.6%	0.019	R
Caucasian	0.0%	0.980	F	0.0%	0.980	F	0.0%	0.991	F	0.0%	0.887	F	0.0%	0.998	F
Chinese	37.5%	0.171	F	0.0%	0.974	F	70.5%	0.009	R	0.0%	0.989	F	66.1%	0.019	R
BC	0.0%	0.787	F	0.0%	0.939	F	0.0%	0.589	F	0.0%	0.857	F	0.0%	0.691	F
CC	0.0%	0.912	F	0.0%	0.932	F	0.0%	0.964	F	0.0%	0.949	F	0.0%	0.939	F
rs6504074	52.4%	0.062	R	39.2%	0.130	F	41.2%	0.116	F	26.2%	0.229	F	55.4%	0.047	R
Caucasian	0.0%	0.967	F	66.7%	0.029	R	0.0%	0.645	F	62.0%	0.048	R	0.0%	0.740	F
Chinese	0.0%	0.567	F	0.0%	0.936	F	0.0%	0.987	F	0.0%	0.935	F	0.0%	0.984	F
OC	0.0%	0.551	F	48.8%	0.142	F	0.0%	0.840	F	45.6%	0.159	F	0.0%	0.794	F
GC	0.0%	0.527	F	0.0%	0.534	F	0.0%	0.486	F	0.0%	0.516	F	0.0%	0.535	F

**Figure 4:**
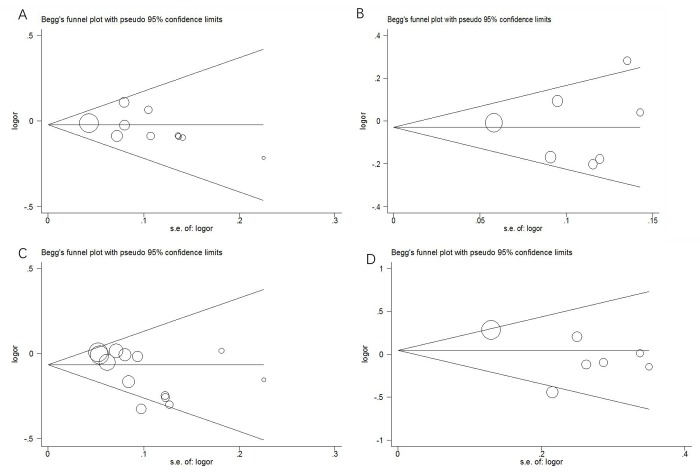
**Funnel plots of publication bias.** (**A**) rs2048718; (**B**) rs4988344; (**C**) rs4986764; (**D**) rs6504074.

In this meta-analysis, we used funnel plots and Egger’s test to estimate publication bias. The funnel plots (Figure 4) failed to discover any prominent asymmetry for the four polymorphisms, which was consistent with the results of Egger’s test (*P* > 0.05). Therefore, we considered that the publication bias in this meta-analysis was not significant.

### Sensitivity analysis

**Figure 5:**
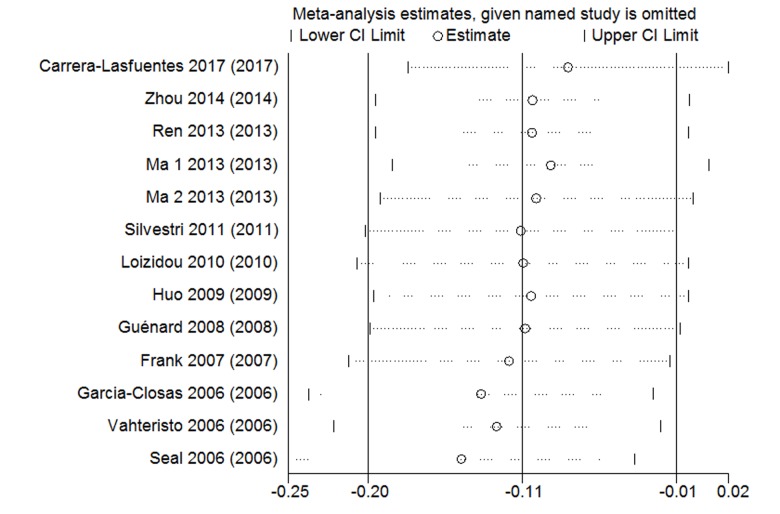
Sensitivity analysis of the associations between rs4986764 polymorphisms and cancer risk.

The sensitivity analysis was used in rs4986764 showed no individual research could alter the pooled ORs significantly (Figure 5), which proved the reliability and credibility of the outcomes.

## DISCUSSION

As a DNA helicase interacts with *BRCA1* directly, *BRIP1* regulates DNA normal double-strand break repair function [1,4]. Germline *BRIP1* mutations, which negatively affect DNA repair and genomic stability, and thus increase the likelihood of cancer development, have been reported to be connected with breast, colon, prostate and ovarian cancer [4,8,12,13]. Recent studies showed that SNPs in genes had an influence on multiple types of cancer [4-6,14]. Numerous previous studies have suggested that *BRIP1* polymorphisms were potentially related to susceptibility of human cancers, especially breast, cervical, and ovarian cancer. However, these published studies reported inconsistent results [7], probably due to the limitations, such as small sample size, mixed ethnic groups, and cancer types. To our knowledge, there are few researches have simultaneously estimated the relationship between common variants in *BRIP1* and the risks of common cancers. With a sample size of 13,716, our meta-analysis showed the overall associations between common polymorphisms of *BRIP1* (rs2048718, rs4988344, rs4986764, and rs6504074) and cancer risk. The pooled analysis found significant association with rs2048718 and rs4986764. And, we found all these 4 SNPs predicted a decreased risk of cancer in Chinese. Moreover, rs6504074 showed an association with gynecologic cancer. And, rs2048718 and rs4986764 polymorphisms showed a decreased risk of cervical cancer.

Compared to other published meta-analyses, we found both consistent and conflicting results. Based on the results of 2 studies in USA and Poland using mouthwash samples, rs4986764 (P919S) had no association with breast cancer [15]. In another meta-analysis, Shi et al reported that rs4986764 may reduce the breast cancer risk for the Caucasian population, especially postmenopausal females who has a family history of breast cancer excluding mutations of BRCA1/2 [16]. However, no association was found in Pabalan’s study [17]. Unfortunately, their meta-analysis failed to analyze the data according to ethnic subgroup. Previous studies failed to find a relation between rs2048718 and rs4988344 polymorphisms and the susceptibility of cervical cancer [5,6,14]. Our study showed that rs4988344 polymorphism has no significant influence on cancer risk among overall population, while subgroup analysis by ethnicity showed a significant association in the Chinese population (Table 3). The most important polymorphisms of *BRIP1* (rs2048718, rs4988344, rs4986764, and rs6504074) were associated with cancer risk among Chinese; however, no significant relationship was observed in Caucasians. We presumed that this discrepancy may have resulted from variations in the genomes of different ethnic groups. These results suggest that ethnic differences and the patient’s environment may play a role in malignancy. On account of the findings of these studies, we estimated the possible association between the 4 SNPs of *BRIP1* and cancer risk. However, *in vivo*, *BRIP1* might be regulated by multiple mechanisms [1,18,19]. We speculated that discrepancy for *BRIP1* in different ethnic groups stems from a diversity regulating mechanism as well.

As to the limitations of this meta-analysis, absence of detailed information and adjusted outcomes should be acknowledged. We failed to considered detailed information like age, sex, lifestyle, and environmental exposures. Besides, we found a decreased risk of rs2048718 and rs4986764 polymorphisms to cervical cancer based on three studies, which needs further well-design multicenter studies including more study subjects to confirm.

## CONCLUSIONS

Overall, this meta-analysis showed that rs2048718 and rs4986764 were associated with a lower cancer risk among overall population. According to the stratified analysis by ethnicity, the rs2048718, rs4988344, rs4986764 and rs6504074 polymorphisms of *BRIP1* were strongly related to cancer susceptibility among Chinese population. And rs6504074 was significant associated with gynecologic cancer. These may made SNPs of *BRIP1* (rs2048718, rs4988344, rs4986764, and rs6504074) be a potential tool for cancer screening and improve early cancer diagnosis.

## MATERIALS AND METHODS

### Search strategy

A profound literature search from PubMed, Web of Science, WanFang, VIP and Chinese National Knowledge Infrastructure (CNKI) databases was conducted up until December 31, 2017, applying the search terms: cancer/tumor/carcinoma/neoplasm, BRIP1, and polymorphism/genotype /SNP. The reference of literature review and eligible articles were also screened for additional relevant publication. Studies conformed to the following criteria were selected: (1) case-control design estimating the relationship of *BRIPI* SNPs and cancer risk; (2) full-text study; (3) all cancer cases confirmed by histopathology, and all cancer-free controls without history of malignant diseases; (4) published in English or Chinese; (5) detailed genotyping data offered. Review papers, not case-control design or studies lack of detailed gene data were excluded. If overlapping cases or controls appeared in two or more different studies, the paper with larger sample size was finally chosen.

### Data extraction

Two reviewers (Liu Di and Wang Meng) reviewed included articles independently. The following information was collected from each included publication: first author, publication year, country or origin, ethnicity, source of control, total number of cases and controls, genotyping methods, genetic distribution of cases and controls group, and P value of Hardy–Weinberg equilibrium (HWE) for controls. Ethnic groups were categorized as Caucasian, Asian, African, and “mixed.” All case and control groups were well controlled. Data with discrepancies were discussed with a senior author until consensus reached.

### Statistical analysis

To measure the associations between *BRIP1* polymorphisms and cancer risk, odds ratio (OR) with 95% conﬁdence interval (CI) was calculated according to the genotypes in cases and controls. The signiﬁcance of the pooled OR was determined by the Z test. All *P* values in this study were two-sided, and a statistic significance was considered if *P* < 0.05. All statistical analyses in our investigation were performed by the software STATA (Version 12.0, Stata Corp, College Station, TX).

The meta-analysis assessed the associations using 5 different genetic models [10,11]: homozygous model (BB vs. AA), heterozygous model (AB vs. AA), dominant model (BB+ AB vs. AA), recessive model (BB vs. AA+ AB), and allele model (B vs. A). “A”, “B” represents the major, minor allele, respectively. Statistical heterogeneity among included studies was evaluated by the Q and I^2^ statistics. Publication bias was accessed with funnel plots and Egger’s test. Sensitivity analysis was conducted to access the statistic stability of polymorphisms including more than 10 studies, by sequentially excluding every individual research and re-checked whether the pooled ORs were changed.
